# *MUC2 *polymorphisms are associated with endometriosis development and infertility: a case-control study

**DOI:** 10.1186/1471-2350-13-15

**Published:** 2012-03-15

**Authors:** Cherry Yin-Yi Chang, Yi Chen, Wu-Chou Lin, Chih-Mei Chen, Chih-Ping Chen, Shan-Chih Lee, Jim Jinn-Chyuan Sheu, Fuu-Jen Tsai

**Affiliations:** 1 Department of Obstetrics and Gynecology, China Medical University Hospital, 2 Yude Road, 40402 Taichung, Taiwan; 2 Institute of Public Health, China Medical University, 91 Hsueh-Shih Road, 40447 Taichung, Taiwan; 3 Human Genetics Center, China Medical University Hospital, 2 Yude Road, 40447 Taichung, Taiwan; 4 Department of Obstetrics and Gynecology, Mackay Memorial Hospital, 92 Sec. 2 Zhongshan Road, 10449 Taipei, Taiwan; 5 School of Medical Imaging and Radiological Sciences, Chung Shan Medical University, 110 Sec.1 Jianguo N. Rd, 40201 Taichung, Taiwan; 6 School of Chinese Medicine, China Medical University, 91 Hsueh-Shih Road, 40447 Taichung, Taiwan; 7 Department of Health and Nutrition Biotechnology, Asia University, 500 Lioufeng Road, 41354 Taichung, Taiwan; 8 School of Post-Baccalaureate Chinese Medicine, China Medical University, 91 Hsueh-Shih Road, 40447 Taichung, Taiwan

## Abstract

**Background:**

Mucins are highly glycosylated proteins protecting and lubricating epithelial surface of respiratory, gastrointestinal and reproductive tracts. Members of the mucin protein family have been suggested to play an important role in development of endometriosis and infertility. This study investigates genetic association of mucin2 (*MUC2*) with the risk of endometriosis and endometriosis-related infertility.

**Methods:**

This case-control study was conducted at China Medical University Hospital, with 195 endometriosis patients and 196 healthy controls enrolled. Genotyping of six SNPs (rs2856111, rs11245936, rs10794288, rs10902088, rs7103978 and rs11245954) within *MUC2 *gene were performed by using *Taqman *genotyping assay; individual SNP and haplotype associations with endometriosis and endometriosis-related infertility were assessed by *χ*^2 ^test.

**Results:**

Endometriosis patients exhibit significantly lower frequency of the rs10794288 C allele, the rs10902088 T allele and the rs7103978 G allele (*P *= 0.030, 0.013 and 0.040, respectively). In addition, the rs10794288 C allele and the rs10902088 T allele were also less abundant in patients with infertility versus fertile ones (*P *= 0.015 and 0.024, respectively). Haplotype analysis of the endometriosis associated SNPs in *MUC2 *also showed significantly association between the most common haplotypes and endometriosis or endometriosis-related infertility.

**Conclusions:**

*MUC2 *polymorphisms, especially rs10794288 and rs10902088, are associated with endometriosis as well as endometriosis-related infertility. Our data present MUC2 as a new candidate involved in development of endometriosis and related infertility in Taiwanese Han women.

## Background

Endometriosis is a common chronic gynecologic disease defined as presence of endometrial tissue outside the uterine cavity, primarily on pelvic peritoneum and ovary. Epidemiology studies reveal that endometriosis affects more than 10% of reproductive age women and possibly causes infertility [1,2]. The prevalence of endometriosis was 0.5-5% in fertile and 25-45% in infertile women [3]. The mechanism underlying endometriosis development remains unclear, even though theories like implantation, altered immunity, and susceptible genetic factors have been proposed to explain the pathogenesis [4-6]. Nevertheless, familial and identical twins studies have established the genetic predisposition to endometriosis development [7].

Clinical manifestation of endometriosis is accompanied by angiogenesis and formation of cellular adhesion [8,9], possibly due to altered peritoneal environment and immune system [10]. In endometriosis patients, changes in levels of growth factors, cytokines and oncofetal antigens may facilitate intraperitoneal endometrial growth and alter the peritoneal environment, which leads to disruption of normal pelvic organ architectures and infertility [8]. For instance, interluekin-1 (IL-1), interleukin-6 (IL-6), interleukin-8 (IL-8), interleukin-10 (IL-10), nuclear factor-κB (NF-κB) and tumor necrosis factor-alpha (TNF-α) are among the major cytokines participating in regulation of immune system, angiogenesis, cell proliferation and tissue invasiveness during the formation of endometriosis [11-14].

Mucins are high molecular weight glycoproteins with function of protecting and lubricating epithelial surface of respiratory, gastrointestinal and reproductive tracts [15]. Mucins are also found to be expressed on activated lymphocytes, supporting the hypothesis that some mucin domains function as cytokines to mediate immune responses [16,17]. Early studies reported that mucin played important role in the progress of tumor invasion, which is influenced by their glycosylation status [15,18-20]. More recently, mucin1 (MUC1) has been reported to be linked to endometriosis and infertility [21,22], and mucin4 (*MUC4*) gene polymorphisms were proved to be associated with endometriosis development as well [23]. The human mucin2 (*MUC2*) gene is located on chromosome 11p15.5, encoding one of the most common gel-forming secreted type of mucin [24]. MUC2 expression was reported to be regulated by many endometriosis-related cytokines, such as IL-1β, IL-6, TNF-α, NF-Kappa B [9,25-27]. Abnormal increase of mucin2 (MUC2) expression was reported to be linked to intestinal and uterine cervix metaplasia progression [28,29].

However, there has been no study yet investigating the relationship between endometriosis and MUC2, while previous functional studies on MUC2 are more focused on its role in the gastrointestinal and respiratory tract [30,31].

The aim of this study was to investigate the possible association of *MUC2 *gene polymorphisms with the risk of endometriosis and endometriosis-related clinical symptoms in a Taiwanese population.

## Methods

### Subjects

In all, 195 patients receiving surgery for ovarian benign disease and pathology-proven endometriosis patients were identified at China Medical University Hospital from 1998-2011 and enrolled in the study. They accepted examination of ultrasound before operation and were diagnosed with ovarian cysts; symptoms of dysmenorrhea, lower abdominal pain, infertility or abnormal menstruation were recorded for each patient. Another 196 healthy controls were recruited from a pool of persons who received regular health checkups at the same hospital. People with ovarian cysts detected by ultrasound or any symptoms of dysmenorrhea, lower abdominal pain, infertility, abnormal menstruation were excluded from the controls. Both patients and controls share similar age profile. Clinical information of patients was collected from medical charts, including clinical stage, lesion size, location, drug treatment and fertility. The definition of endometriosis staging was based on criteria of the American Society for Reproductive Medicine: stage 1, minimal; stage 2, mild; stage 3, moderate; and stage 4, severe [32]. Individual leukocytes were drawn by vein-puncture and separated by Ficoll-paque (GE, Uppsala, Sweden) for genomic DNA isolation. Signed consent was obtained from each study subject, approved by the Institutional Review Board at China Medical University Hospital.

### Genotyping

Genomic DNA was extracted from peripheral blood leukocytes according to standard protocol (Genomic DNA kit; Qiagen, Valencia, CA, USA). DNA fragments containing target SNP sites were amplified by PCR using the *Taqman *SNP genotyping assay system from Applied Biosystems, Inc. (Carlsbad, CA, USA). The probe IDs for these SNP sites were selected from the ABI SNP genotyping databank http://www.allsnps.com and listed in Additional file 1: Table S1. PCR amplification conditions consisted of initial denaturation at 95°C for 5 min, followed by 40 cycles of 95°C for 10 sec, and 56°C for 10 sec. Genetic variations were detected by reading the fluorescence signals of PCR products. A positive fluorescent signal indicates a perfect match between the probe and the tested DNA, thus identifying the allele type.

### Statistical analysis

Allelic and genotypic frequency distributions for these SNP sites in endometriosis patients and controls were performed by *χ*^2 ^analysis using SPSS software (version 10.0, SPSS Inc. Chicago, Illinois, US). Allelic and genotypic frequencies are expressed as percentages of the total number of alleles and genotypes. Odds Ratios (ORs) were calculated for allelic and genotypic frequencies with 95% confident interval (95% CI). Adherence to the Hardy-Weinberg equilibrium constant was confirmed by *χ*^2 ^test with one degree of freedom by PLINK program [33].

Haplotype association was analyzed using Bayesian statistical method available in the program Phase 2.1 [34]. Lewontin's coefficient D' and the linkage disequilibrium (LD) were determined between each pairs of biallelic loci using absolute association (*r*^2^) [35]. Haploview 4.2 (Whitehead Institute for Biomedical Research, Cambridge, MA) was used to examine the structure of the LD block [36]. A *p *value of less than 0.05 was considered statistically significant.

### Functional analyses and secondary structure prediction

Functional characterization and annotation of MUC2 were performed by aligning the sequence with functional motifs in PROSITE protein domain database [37]. NetSurfP ver. 1.1 was used to predict the secondary structure and surface accessibility of MUC2 [38] Relative and absolute surface accessibility were also calculated for each residue. 

## Results

### *MUC2 *polymorphisms and endometriosis

**Figure 1:**
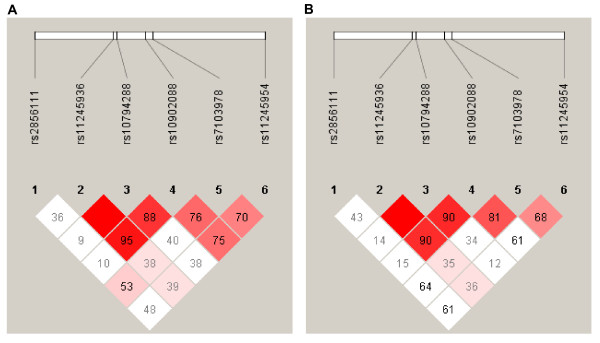
**Pairwise linkage disequilibrium (LD) between SNPs of the MUC2 gene**. LD maps were shown for controls (A) and patients (B). Values shown are for D'.

To test whether SNPs in *MUC2 *gene play role in endometriosis development, a total of six SNPs were selected for this study, with minor allelic frequencies over 4% in Chinese Han population based on the information in international HapMap project databank http://www.hapmap.org (Additional file 1: Table S1). These SNPs and their LD map were shown in Figure 1A. Genotype and allele frequencies were summarized in Table 1 for both the patient and the control groups. Allele distribution analyses revealed significant associations between endometriosis and genetic variations at three of the six SNPs (Table 1). Endometriosis patients had significantly lower frequencies of C allele at rs10794288, T allele at rs10902088 and G allele at rs7103978, as compared to the controls (*P *= 0.030, 0.013 and 0.040, respectively) (Table 1). Genotype analysis indicated that frequency of the TT genotype at rs10902088 was significantly lower in patients than in controls (*P *= 0.045; OR = 0.47, 95% CI: 0.27-0.83), while the CC genotype at rs10794288 and the AG genotype at rs7103978 had protective effect against endometriosis (OR = 0.56 and 0.47, respectively; 95% CI < 1) (Table 1). These results implied possible relationship between three individual *MUC2 *polymorphisms and endometriosis development.

**Table 1:** Association between SNPs in MUC2 gene and endometriosis in Taiwanese patients and controls

SNP	Genotype/allele	No. (%) of patients		No. (%) of controls		*p*-value^a^	OR	95% CI
rs2856111	CC	35	(18.5)	35	(18.2)	0.29	0.79	0.46-1.36
	CT	96	(50.8)	111	(57.8)		0.69	0.43-1.10
	TT	58	(30.7)	46	(24.0)		1.00	
	C	166	(43.9)	181	(47.1)	0.37	0.88	0.66-1.17
	T	212	(56.1)	203	(52.9)		1.00	
rs11245936	AA	1	(0.5)	3	(1.6)	0.34	0.32	0.03-3.10
	AG	21	(11.0)	28	(14.5)		0.72	0.39-1.32
	GG	169	(88.5)	162	(83.9)		1.00	
	A	23	(6.0)	34	(8.8)	0.14	0.66	0.38-1.22
	G	359	(94.0)	352	(91.2)		1.00	
rs10794288	CC	34	(17.4)	45	(23.0)	0.092	0.56	0.33-0.96
	CT	87	(44.6)	96	(49.0)		0.67	0.43-1.06
	TT	74	(37.9)	55	(28.0)		1.00	
	C	155	(39.7)	186	(47.4)	0.030*	0.73	0.55-0.97
	T	235	(60.3)	206	(52.6)		1.00	
rs10902088	TT	27	(14.0)	43	(22.3)	0.045*	0.47	0.27-0.83
	CT	93	(48.2)	95	(49.2)		0.74	0.47-1.16
	CC	73	(37.8)	55	(28.5)		1.00	
	T	147	(38.1)	181	(47.4)	0.013*	0.70	0.52-0.93
	C	239	(61.9)	205	(52.6)		1.00	
rs7103978	GG	2	(1.0)	1	(0.5)	0.053	1.84	0.16-21.58
	AG	19	(9.8)	37	(18.9)		0.47	0.26-0.85
	AA	172	(89.1)	158	(80.6)		1.00	
	G	23	(6.0)	39	(9.9)	0.040*	0.57	0.34-0.98
	A	363	(94.0)	353	(90.1)		1.00	
rs11245954	GG	0	(0.0)	0	(0.0)	NA	NA	NA
	AG	21	(10.8)	26	(13.5)		0.78	0.42-1.43
	AA	173	(89.2)	166	(86.5)		1.00	
	G	21	(5.4)	26	(6.8)	0.43	0.79	0.44-1.43
	A	367	(94.6)	358	(93.2)		1.00	

*Indicates statistical significance

^a^*P*-values were calculated using *χ*^2 ^test without corrections for multiple test

### *MUC2 *polymorphisms and infertility

Because endometriosis was suggested as one source of female infertility, we asked whether genetic variations in *MUC2 *play roles in this process. The patients were subgrouped into patients with infertility or without infertility for genotype and allele distribution analyses. Patients without sexual experience were excluded in this study. As shown in Table 2, the C allele at rs10794288 and the T allele at rs10902088 were much less prevalent in patients with infertility (*P *= 0.015 and 0.024, respectively). The TT genotype at rs10902088 were absent in the infertile patient group, and genotype distribution was also significantly different between the infertile and the fertile patients for this SNP (*P *= 0.047, data not shown). This finding revealed protective potential of these two genetic variations in *MUC2 *against infertility in endometriosis patients.

**Table 2:** Association between allele distributions of SNPs in MUC2 and endometriosis-related infertility

SNP	Infertile MAF	Non-Infertile MAF	*P*-value^a^	OR	95% CI
rs2856111	48.1	41.3	0.36	1.32	0.73-2.38
rs11245936	3.8	5.4	0.64	0.70	0.16-3.16
rs10794288	24.1	41.7	0.015*	0.44	0.23-0.87
rs10902088	26.0	43.1	0.024*	0.46	0.24-0.91
rs7103978	5.8	5.8	0.99	0.99	0.28-3.54
rs11245954	3.8	4.7	0.79	0.82	0.18-3.72

*Indicates statistical significance

*MAF *minor allele frequency

^a^*P*-values were calculated using *χ*2 test

### Haplotype analysis of *MUC2 *polymorphisms

The endometriosis-related SNPs (rs10794288, rs10902088 and rs7103978) found in individual tests were selected for haplotype analysis of endometriosis and disease-related clinical symptoms. Association data were enlisted in Table 3 for all the haplotypes with frequency higher than 1% presented in the case or control group. As shown in Table 3, the two most common haplotypes (T-C and C-T) of rs10794288 and rs10902088 were significantly associated with both endometriosis and infertility development in the patients. Haplotype T-C was more common in endometriosis patients (*P *= 0.012) and patients with infertility (*P *= 0.0091) (Table 3). By contrast, haplotype C-T might be a protective factor against endometriosis (*P *= 0.035) and endometriosis-related infertility (*P *= 0.025) (Table 3). Similarly, the most frequent haplotype T-C-A of rs10794288, rs10902088 and rs7103978 was significantly associated with both endometriosis (*P *= 0.0063) and endometriosis-related infertility (*P *= 0.0066) as a risk factor of the disease (Table 3). In the patient group, LD map demonstrated higher linkage between these three SNPs (Figure 1B) compared to the control group (Figure 1A), suggesting a genetic relationship between these SNPs and endometriosis. Therefore, haplotypes of these SNPs in *MUC2 *gene could serve as an indicator of susceptibility to endometriosis and endometriosis-related infertility.

**Table 3:** Association between MUC2 haplotypes and endometriosis or endometriosis-related infertility

SNP marker	Haplotype	Endometriosis			Endometriosis-related infertility		
		Case (%)	Control (%)	*P*-value^a^	Infertility (%)	Non-infertility (%)	*P*-value^a^
rs10794288, rs10902088	T-C	58.6	49.6	0.012*	74.9	57.2	0.0091*
	C-T	35.8	43.2	0.035*	23.4	38.3	0.025*
	C-C	3.4	3.7	0.83	0.1	2.6	0.20
	T-T	2.1	3.5	0.27	1.6	1.9	0.89
rs10794288, rs10902088, rs7103978	T-C-A	57.8	48.1	0.0063*	74.8	56.3	0.0066*
	C-T-A	32.0	35.9	0.25	22.0	34.1	0.06
	C-T-G	3.8	7.2	0.036*	1.4	4.2	0.29
	C-C-A	3.4	3.7	0.81	0	2.6	0.20
	T-T-G	1.5	2.0	0.61	1.6	1.1	0.76

*Indicates statistical significance. aP-values were calculated using χ2 test

### *MUC2 *polymorphisms and amino acid substitutions

One of the endometriosis-associated SNPs (rs10902088) in *MUC2 *gene caused an amino acid substitution (Additional file 1: Table S1), and functions of MUC2 might be altered if such substitution changes surface charge, protein stability or folding. This SNP located at amino acid Asn1149, which was predicted to be within a long coil region. The relative surface accessibility of Asn1149 was estimated to be 0.707 with reference to a fully exposed side chain, representing a highly exposed residue. Genetic variation of rs10902088 introduced an asparagine to lysine substitution, which was well fit in this position as a highly exposed residue, but the surface charge at this region would be reversed. Accordingly, this substitution was more likely to disrupt inter- or intra-molecular interactions of MUC2 rather than protein stability. On the other hand, rs10794288 and rs7103978 were silent mutations, which only influence codon usages instead of amino acid substitutions.

## Discussion

Mucin proteins were known to be heavily glycosylated, on which oligosaccharide structures turned to be tumor-associated antigens and are essential for antibody recognition [39-41]. Our data revealed two *MUC2 *polymorphisms (rs10794288 and rs10902088) were associated with endometriosis development and the related infertility. Polymorphism at rs10902088 generates an amino-acid change Asn1149Lys, while rs10794288 is a silent substitution. Although Asn1149 is not a typical site for N-linked glycosylation, this substitution to positively charged residue may influence the glycosylation states of several neighboring serines and Asn1154, which is within a typical N-linked glycosylation tripeptide sequon Asn-Ile-Ser [42]. Therefore, this polymorphism may alter the glycosylation status of MUC2, which may subsequently influence the interaction between MUC2 and host environments.

Endometriosis development is associated with altered inflammatory and immune responses, while clinical feature of endometriosis also mimics malignant reproductive disease, such as progressive invasion to adherent pelvic organ and recurrence abilities. Mucins are secreted by epithelium cells of reproductive tissues, generating the mucus of cervix and endometrium, which plays an important role in reproductive physiology. Impaired mucin secretion could impede spermatozoa migration, which may contribute to female infertility. Unlike the less consistent expression patterns of MUC1, MUC6 and MUC5AC in normal and cancer tissues, MUC2 levels were always measured low in normal endometrial and cervical tissue, and elevated MUC2 expressions were specifically found in various neoplastic lesions [28,29,43-45]. However, expression patterns of MUC2 in ovarian tumor were heterogenic [18]. Boman et al. reported that MUC2 were mainly present in benign and borderline ovarian tumor [46], while Dong et al. showed that breast cancer patients with presence of MUC2 expression had shorter disease-free survival [47]. We found that the minor allele of rs7103978 decreases the cognate codon frequency from 15.8‰ to 7.8‰ (Kazusa DNA Res. Inst. http://www.kazusa.or.jp/codon/), which may increase the odds of premature translation termination and thus reduce MUC2 level. Therefore, our result suggested that expression of MUC2 may facilitate cell invasion or proliferation abilities. The observed association of *MUC2 *polymorphisms and endometriosis may help us further elucidate the link between endometriosis and certain subtypes of ovarian cancer, if such genetic alterations were also present in the ovarian cancer patients.

Although endometriosis could cause pelvic adhesion and tubal occlusion which lead to infertility, some patients without anatomic disruption still had the problem of impaired fertilization. Possible mechanisms of endometriosis-related infertility include impaired folliculogenesis induced by abnormal immunological, chemical factors or toxins, poor oocyte quality, inhibited binding of spermatozoa to the zona-pellucida and impaired implantation of embryo. This phenomenon was correlated to changes of cytokines and growth factors in endometrium, follicular fluid and peritoneal fluid [8]. Previous studies have already showed the positive association of endometriosis and polymorphism of cytokine genes [7,48]. Li et al. showed that macrophage induced IL-6 up-regulated the MUC1 but down-regulated MUC2 expression [9]. Up-regulation of MUC1 was associated with implantation failure [22]. IL-1 was also found to up-regulate MUC2 expression, and IL-1 was thought to regulate immune and inflammation response in endometrium and modulate extracellular matrix modeling of endometrium during menstruation and implantation [49]. Moreover, previous studies demonstrated that IL-8, TNF-α and NF-κB tend to increase in the peritoneal fluid according to the severity of dysmenorrhea, extent pelvic adhesion and proliferation of endometrial stroma cells, and MUC2 expression could increase accordingly via activation of NF-κB pathway through these cytokines [50-52]. Therefore, it is plausible that altered level of MUC2 could affect fertility as a downstream effecter that can further influence the secretion of mucus, sperm motility, oocyte quality and receptivity of endometrium.

## Conclusions

In this study, our data revealed a significant association between *MUC2 *polymorphisms and endometriosis in a Taiwanese population. The results imply that MUC2 may play a role in the pathogenesis of endometriosis and endometriosis-related infertility, while the mechanisms underlying this phenomenon remain to be elucidated. As a major secreted form of mucins, MUC2 may have the ability to affect more surrounding tissues than the membrane-bound form of mucins. Since MUC2 is not as well investigated as some other mucins in reproductive organs, its molecular function in endometriosis and infertility is worth future study.

## Competing interests

The authors declare that they have no competing interests.

## Authors' contributions

CY-YC and YC: study design, execution and manuscript drafting; YC and C-MC: statistical analysis; CY-YC, W-CL and F-JT: patient collection; C-PC, S-CL and JJ-CS: critical discussion and manuscript editing. All authors read and approved the final manuscript.

## Pre-publication history

The pre-publication history for this paper can be accessed here:

http://www.biomedcentral.com/1471-2350/13/15/prepub

## Supplementary Material

Additional file 1 **Table S1**. Probes been used for SNPs in MUC2 gene. Click here for file
