# Supplementary material for: MUC2 polymorphisms are associated with endometriosis development and infertility: a case-control study
Source: BMC Med Genet. 2012 Mar 15;13:15. doi: 10.1186/1471-2350-13-15 (PMC3338096; doi:10.1186/1471-2350-13-15)
Supplement: Additional file 1 — Table S1. Probes been used for SNPs in MUC2 gene. [file 1471-2350-13-15-S1.DOC]

# Additional files

### Supplementary Table 1. Probes been used for SNPs in MUC2 gene

| SNP | Allele frequencies^a^ | | Amino acid change | ABI probe assay ID |
| --- | --- | --- | --- | --- |
| rs2856111 | T:45% | C:55% | Leu58Pro | C___1841494_10 |
| rs11245936 | G:95% | A:5% | Ser832Gly | C__25652316_10 |
| rs10794288 | T:60% | C:40% | Asp872Asp | C__25652351_10 |
| rs10902088 | C:58% | T:42% | Asn1149Lys | C____206941_20 |
| rs7103978 | A:94% | G:6% | Ala1200Ala | C__31856643_10 |
| rs11245954 | A:95% | G:5% | Ser2459Cys | Muc2_5945^b^ |

^a^Allele frequencies listed are from Han Chinese population distribution data in the HapMap database.

^b^Custom designed probe.
